# Cholera Toxin Production in *Vibrio cholerae* O1 El Tor Biotype Strains in Single-Phase Culture

**DOI:** 10.3389/fmicb.2020.00825

**Published:** 2020-05-05

**Authors:** Yeongjun Baek, Donghyun Lee, Jiwon Lee, Youngbae Yoon, G. Balakrish Nair, Dong Wook Kim, Eun Jin Kim

**Affiliations:** ^1^Department of Pharmacy, College of Pharmacy, Hanyang University, Ansan, South Korea; ^2^Institute of Pharmacological Research, Hanyang University, Ansan, South Korea; ^3^Microbiome Laboratory, Rajiv Gandhi Centre for Biotechnology, Thiruvananthapuram, India

**Keywords:** *Vibrio cholerae*, cholera, biotype, cholera toxin, El Tor biotype

## Abstract

*Vibrio cholerae* O1 serogroup strains have been classified into classical and El Tor biotypes. Cholera, a life-threatening diarrheal disease, can be caused by either biotype through the cholera toxin (CT) that they produce. To increase our knowledge of the pathogenicity of bacteria, we must understand the toxigenicity of bacteria. CT production by classical biotype strains in simple single-phase cell cultures has been established; however, special culture media and growth conditions that are not appropriate for mass production of CT are required to facilitate CT production in El Tor biotype strains. In this report, we produced CT in El Tor biotype strains using simple media and single-phase culture conditions. A single point mutation in ToxT, a transcriptional activator of toxin co-regulated pilus (TCP) and CT, enabled the El Tor biotype strains to produce CT in similar quantities as classical biotype strains in single-phase laboratory culture conditions. CT production capacity varied between El Tor biotype strains. Wave 2 and 3 atypical El Tor strains tended to produce more CT than prototype Wave 1 strains. Wave 2 and 3 strains lack neutral fermentation; however, the capacity for neutral fermentation was not associated with significant differences in CT production by El Tor biotype strains. The Wave 3 strain that caused the 2010 cholera outbreak in Haiti produced CT only when neutral fermentation was abolished. The disparity in CT production between the seventh cholera pandemic strains highlight the differences in virulence between strains and the cause of population changes in *V. cholerae*.

## Introduction

Serogroup O1 and O139 *Vibrio cholerae* strains cause a fatal diarrheal disease, cholera (Kaper et al., 1995). The symptoms of cholera are induced primarily by the cholera toxin (CT) that is produced by *V. cholerae* bacteria. The CT gene *ctxAB* is carried by a filamentous phage, CTXΦ, which can be integrated into both chromosomes in *V. cholerae* (Waldor and Mekalanos, 1996; Kim et al., 2017). Two biotypes of *V. cholerae* O1 serogroup strains—classical and El Tor—have been recognized as the causative agents of the first to sixth and the seventh cholera pandemics, respectively (Safa et al., 2010; Kim et al., 2015).

Classical biotype strains produce classical biotype-specific CT, whereas El Tor biotype strains synthesize El Tor biotype-specific CT (Raychoudhuri et al., 2009). The 2 toxins differ by 2 amino acids at positions 39 and 68 in the binding subunit (CTB) of the toxin, but the amino acid sequences are identical in the active subunit (CTA). Recently, atypical El Tor strains that produce classical biotype CT have replaced prototype El Tor strains globally (Kim et al., 2015).

Cholera is caused by the CT that is produced by and secreted from the bacteria in the intestinal environment of human hosts; however, inducing CT production *in vitro* is challenging. Laboratory conditions for stimulating CT production in classical biotype strains have been established, whereas efforts to establish CT production in El Tor biotype strains *in vitro* have demonstrated limited success (Ghosh-Banerjee et al., 2010). Culture in media that contain bicarbonate stimulates CT production in El Tor biotype strains (Iwanaga and Yamamoto, 1985; Abuaita and Withey, 2009). An unusual bi-phasic growth condition—described as AKI conditions—has been created for El Tor biotype strains, in which the bacterial culture is kept under static conditions without shaking for 4 h, followed by vigorous shaking for 16 h in AKI media (Iwanaga et al., 1986). However, monitoring CT production and especially, mass production of CT by El Tor biotype strains remains difficult when using these methods.

Recently, we constructed El Tor biotype strains that can be transduced by CTXΦ by inducing the expression of CTX phage receptor, toxin co-regulated pilus (TCP) (Kim et al., 2017). These El Tor strains were constructed by introducing a point mutation in the ToxT transcription factor, which regulates the expression of TCP and CT (Childers et al., 2011). In *V. cholerae* strains, ToxT typically contains a tyrosine at position 139 (139Y) and does not induce CT and TCP under conventional laboratory conditions; however, CT and TCP expression in El Tor biotype strains has been affected under laboratory conditions when the Tyr-139 is replaced by phenylalanine (139F) (Kim et al., 2017). CT production in El Tor strains that contain the *toxT*-139F allele was briefly demonstrated under agglutinating conditions (culture at 30°C in media that was adjusted to pH 6.5 with 50 mM Tris buffer), which had been established for classical biotype strains (Davis et al., 1999). Although CT production by classical biotype strains has been optimized under agglutinating conditions, we expected El Tor biotype strains to have different optimal conditions for CT production.

In this study, we determined the optimal conditions for CT production by El Tor biotype strains that contain the *toxT*-139F allele by varying the pH of the culture medium and growth temperature. CT production in El Tor strains varied, depending on the strain: CT production in prototype (Wave 1) El Tor biotype strains was induced by the *toxT*-139F allele but was less than 50% of that in classical biotype strains; atypical El Tor strains (Wave 2 and 3) synthesized more CT than Wave 1 strains, with several Wave 2 strains producing 200% more toxin than classical biotype strains under agglutinating conditions. Atypical El Tor strains produced CT at 30°C and 37°C, whereas 30°C was more favorable for toxin production in classical and Wave 1 El Tor biotype strains.

We also constructed a derivative of the classical biotype strain that contained the *toxT*-139F allele to confirm that this allele also enhances toxin production in classical biotype strains. When compared with CT production by classical biotype strains under agglutinating condition, the engineered classical biotype strain generated 400% more CT in regular LB media and under agglutinating conditions.

Although the CT production condition that we have described in this report might not reflect the physiological conditions in the human host directly, our results provide insights into the pathogenicity of *V. cholerae*.

## Materials and Methods

### Bacterial Strains and Bacterial Culture

The bacterial strains used in this study are listed in Table 1.

**TABLE 1:** *V. cholerae* strains used in this study.

**strain**	***toxT* genotype**	**VC1589 genotype**	***ctxB* type**	**Reference and genome sequence information**
**Classical biotype strain**				
O395	139Y*	T7^#^	Classical (*ctxB1*)	CP000626/CP000627 (Mutreja et al., 2011)
YJB001	139F	T7	Classical (*ctxB1*)	
**Wave 1 El Tor biotype strains**				
N16961	139Y	T7	El Tor (*ctxB3*)	AE003852/AE003853 (Heidelberg et al., 2000)
YJB002	139Y	T6	El Tor (*ctxB3*)	
YJB003	139F	T7	El Tor (*ctxB3*)	
YJB004	139F	T6	El Tor (*ctxB3*)	
T19479	139Y	T7	El Tor (*ctxB3*)	ERS013250 (Mutreja et al., 2011)
YJB005	139Y	T6	El Tor (*ctxB3*)	
YJB006	139F	T7	El Tor (*ctxB3*)	
YJB007	139F	T6	El Tor (*ctxB3*)	
**Wave 2 El Tor biotype strains**				
B33	139Y	T6	Classical (*ctxB1*)	ACHZ00000000 (Faruque et al., 2007)
YJB008	139Y	T7	Classical (*ctxB1*)	
YJB009	139F	T6	Classical (*ctxB1*)	
YJB010	139F	T7	Classical (*ctxB1*)	
MJ1236	139Y	T6	Classical (*ctxB1*)	CP001485/CP001486 (Grim et al., 2010)
YJB011	139Y	T7	Classical (*ctxB1*)	
YJB012	139F	T6	Classical (*ctxB1*)	
YJB013	139F	T7	Classical (*ctxB1*)	
MG116025	139F	T6	El Tor (*ctxB3*)	ERS013135 (Mutreja et al., 2011)
YJB014	139F	T7	El Tor (*ctxB3*)	
YJB015	139Y	T6	El Tor (*ctxB3*)	
**Wave 3 El Tor biotype strains**				
IB4122	139Y	T6	Classical (*ctxB1*)	ERS013264 (Nguyen et al., 2009)
YJB016	139Y	T7	Classical (*ctxB1*)	
YJB017	139F	T6	Classical (*ctxB1*)	
YJB018	139F	T7	Classical (*ctxB1*)	
IB5230	139Y	T6	Haitian (*ctxB7*)	AELH00000000.1 (Chin et al., 2011)
YJB019	139Y	T7	Haitian (*ctxB7*)	
YJB020	139F	T6	Haitian (*ctxB7*)	
YJB021	139F	T7	Haitian (*ctxB7*)	

*139Y: amino acid position 139 in *toxT* is Tyr, 139-F: amino acid position 139 in *toxT* is Phe. ^#^T7: VC1589 contains 7 thymines from nucleotides 309 to 315 and encodes functional α-acetolactate decarboxylase. T6: VC1589 contains 6 thymines at this position and encodes a truncated product.

### *toxT* Allele Exchange

Two 843 bp DNA fragments, encompassing the 50 nucleotides upstream of the translation start codon to nucleotide 793 of *toxT*, were amplified by PCR using primers the toxT-XbaIF: CCG GCC TCT AGA TAC GTG GAT GGC TCT CTG CG and toxT-SacIR: CCG GCC GAG CTC CAC TTG GTG CTA CAT TCA from strains MG116025 and N16961, respectively. The fragments were inserted into a suicide plasmid, pCVD442. The SNP at nucleotide 416 (A416 in N16961 and T416 in MG116025) lies in the center of these fragments. The *toxT*-139F allele of MG116025 was replaced by the *toxT*-139Y allele of N16961 by allelic exchange, and similarly, the *toxT*-139Y alleles of other strains were replaced with the *toxT*-139F allele of MG116025 (Donnenberg and Kaper, 1991).

### VC1589 Allele Exchange

Using the VC1589-XbaIF (CGG TCT AGA CGG CGG GCG TCA ACT CAA CG) and VC1589-SacIR (CGG GAG CTC TTG GGC GAT AAG TTG TGC TC) primer set, a 1,261-bp fragment that encompassed the functional VC1589 allele from the N16961 Wave 1 El Tor biotype strain and a 1,260-bp fragment that contained the nonfunctional VC1589 allele from the B33 Wave 2 atypical El Tor strain were amplified by PCR and subcloned into the suicide plasmid pCVD442 to generate pCVD442-VC1589-T7 and pCVD442-VC1589-T6, respectively. pCVD442-VC1589-T7 was conjugally transferred to Wave 2 and 3 atypical El Tor strains to replace their nonfunctional VC1589 with a functional VC1589 allele. Similarly, functional VC1589 in Wave 1 El Tor biotype strains was replaced by allelic exchange using pCVD442-VC1589-T6. The replacement of the VC1589 allele in each strain was confirmed by sequencing.

### Measurement of Cholera Toxin Production

The GM1 enzyme-linked immunosorbent assay was used to determine the CT production in culture supernatant (Lee et al., 2012). Purified whole CT (Cayman Chemical, Ann Arbor, MI, United States) was used to provide a standard curve. Rabbit polyclonal anti-CTB (GeneTex, Irvine, CA, United States) and anti-rabbit IgG (GeneTex) were used for detection. The optimal laboratory conditions for CT production from classical biotype strains were as follows: culture at 30°C, LB pH 6.5 buffered with 50 mM Tris–which has been described as “agglutinating condition” (Waldor and Mekalanos, 1996). CT production (amount of CT/1 OD_600_) in the classical biotype strain O395 under such conditions was considered the reference CT production value. CT production from El Tor strains was measured in culture media after 16 h of single-phase shake culture at 30 and 37°C in LB, PBS-buffered LB, AKI media (0.5% NaCl, 0.3% NaHCO_3_, 0.4% yeast extract, and 1.5% Bacto-Peptone), and LB buffered with 50 mM Tris pH 6.5. CT production (amount of CT/1 OD_600_) values of mean ± standard deviation in El Tor strains were obtained from three independent experiments and were compared with the reference value. Expression of CT in *V. cholerae* strains was additionally confirmed by immunoblot analysis using anti-CT (Supplementary Figure S1).

## Results

### Cholera Toxin Production in Wave 1 El Tor Biotype Strains

**FIGURE 1:**
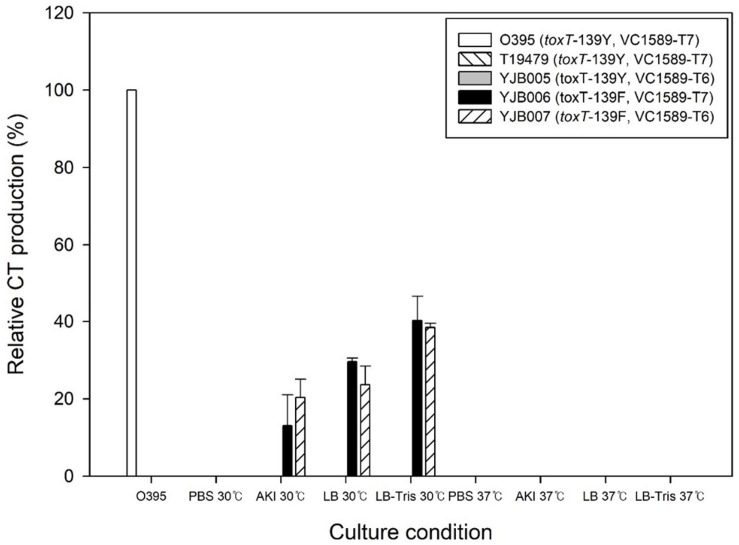
CT production in the Wave 1 strain T19479 and its derivatives. Wave 1 strains primarily contain *toxT*-139Y and functional VC1589 (VC1589-T7) alleles. Derivatives of T19479 (YJB006 and YJB007), which contain the *toxT*-139F allele, produced CT in single phase cultures at 30°C in amounts equivalent to approximately 20–40% of the reference value (CT production from classical biotype strain O395 in 30°C in Tris–buffered LB). However, these strains did not produce CT at 37°C. The following media were used: PBS; PBS-buffered LB, AKI; AKI media, LB-Tris; LB buffered with 50 mM Tris pH 6.5.

Cholera toxin production by various El Tor biotype strains was compared with that of O395 under agglutinating conditions (reference CT production). *V. cholerae* strains usually contain the *toxT*-139Y allele (1 strain, MG116025, reportedly contains the *toxT*-139F allele), and no CT is produced in El Tor strains under single-phase laboratory shake culture conditions (Kim et al., 2017). We confirmed that regardless of the culture medium, no measurable CT was produced in El Tor strains that carry the *toxT*-139Y allele, except for 1 Wave 3 strain, described below. On replacing the *toxT*-139Y allele with *toxT*-139F in a Wave 1 El Tor biotype strains—T19479-*toxT*-139F, cultured under agglutinating conditions—CT production was detected (Figure 1), albeit at levels that were less than 40% versus the classical biotype strain, and no CT was produced at 37°C. The function of the neutral fermentation gene (VC1589) did not influence CT production in this strain (Figure 1).

The other Wave 1 El Tor biotype strain, N16961, which produces CT in amounts that are comparable with classical biotype strains under AKI conditions, did not generate CT in any medium under single-phase culture conditions, despite the *toxT*-139Y allele being replaced by *toxT*-139F (Supplementary Figure S2). Based on these results, we determined that Wave 1 El Tor biotype strains can be manipulated to produce CT in single-phase culture.

### Cholera Toxin Production in Wave 2 Strains

**FIGURE 2:**
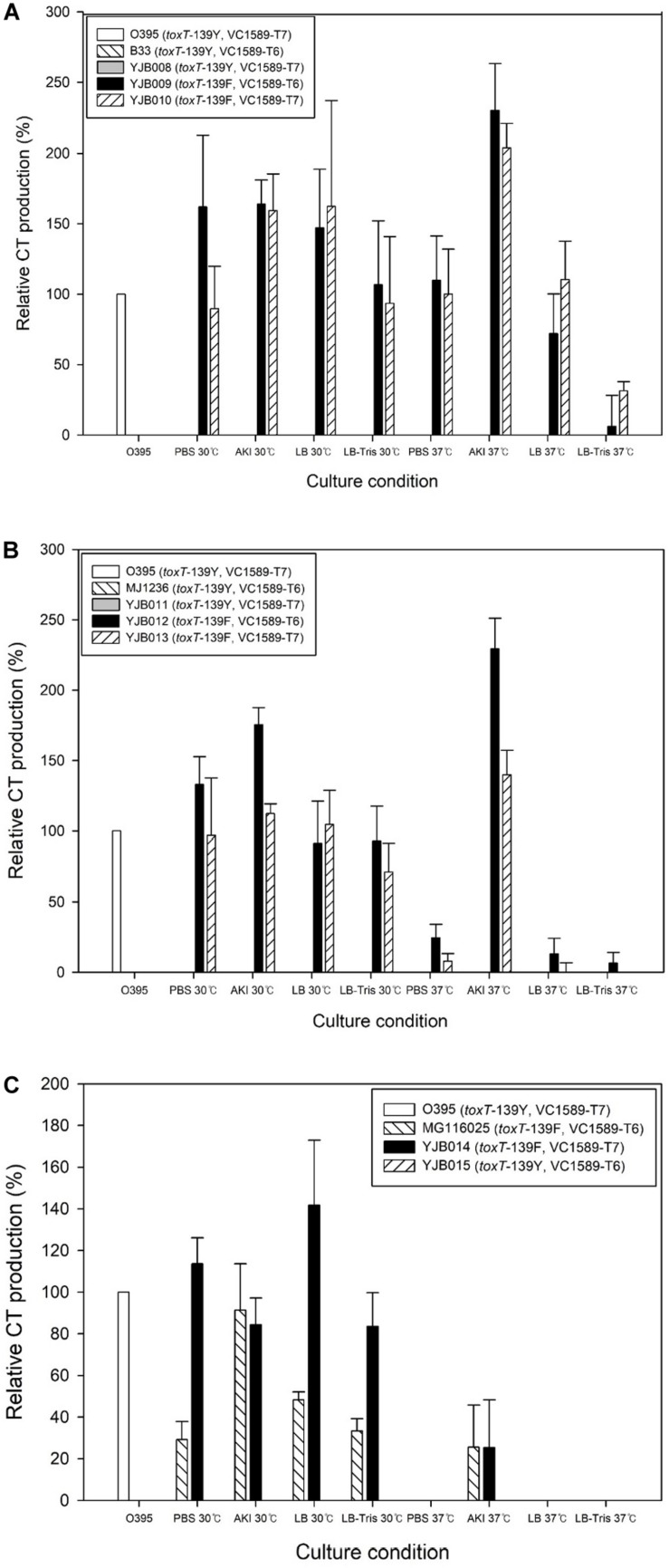
CT production in Wave 2 strains and their derivatives. **(A)** Strain B33 and its derivatives. B33 and its derivative, YJB008, which contains the *toxT*-Y allele, did not produce CT, whereas the B33 derivatives YJB009 and YJB010 that contained the *toxT*-139F allele produced CT at up to 200% of the reference value. **(B)** CT production in the MJ1236 strain and its derivatives was similar to B33 and its derivatives. **(C)** CT production in the MG116025 strain and its derivatives. MG116025 contained the *toxT*-139F allele and produced CT under laboratory conditions. No detectable CT was produced in a derivative of MG116025 (YJB015) that contained the *toxT*-139Y allele. CT produced in MG116025 is the El Tor type (*ctxB3*), whereas CT produced from two other Wave 2 strains is the classical type (*ctxB1*). A notable difference in CT production in the Wave 2 strains B33 and MJ1236 compared with Wave 1 strains is that the former produced CT at 37 and 30°C.

Three Wave 2 El Tor strains that harbored the *toxT*-139Y allele—B33, MJ1236, and MG116025—were tested with regard to their CT production. None of these strains generated CT under conditions tested in this study (Figure 2).

#### Derivatives of B33 That Contain the *toxT*-139F Allele

B33 (*toxT*-139Y, VC1589-T6) has a tandem repeat of CTX-2 on chromosome 2. YJB009 (*toxT*-139F, VC1589-T6) and YJB010 (*toxT*-139F, VC1589-T7) generally produced as much CT as classical biotype strains in the media that were tested. In cultures in AKI media at 37°C, CT production was approximately twice that of the O395 classical biotype strain. In the same medium, slightly more CT was synthesized at 37°C than 30°C. The function of the V1589 gene (nonfunctional VC1589 in YJB009 and functional VC1589 in YJB010) did not affect CT production significantly (Figure 2A). The CT that was produced from these strains was of the classical type, because the CTX-2 in them contains the classical type *ctxB* allele (*ctxB1*).

#### Derivatives of MJ1236 That Contain the *toxT*-139F Allele

MJ1236 is closely related to B33, although these strains were isolated in Bangladesh and Mozambique, respectively. They contain the same CTX phage array (a tandem repeat of CTX-2 on chromosome 2). When cultured at 30°C, CT production by YJB012 (*toxT*-139F, VC1589-T6) and YJB013 (*toxT*-139F, VC1589-T7), which contained *toxT*-139F allele, was similar to the reference CT production, regardless of culture medium (Figure 2B). The function of VC1589 (nonfunctional VC1589 in YJB012 and functional VC1589 in YJB013) did not influence CT production significantly in these strains. At 37°C in AKI media, CT production by YJB012 was approximately two-fold that of the reference strain under agglutinating conditions.

#### MG116025

The original MG116025 strain contains the *toxT*-139F allele. We constructed a derivative of MG116025, YJB015, that contained *toxT*-139Y and VC1589-T6 to confirm that native MG116025 produces CT but the strain contained *toxT*-139Y allele does not (Figure 2C). Another derivative of MG116025 that contained *toxT*-139Y and functional VC1589 did not produce CT either (data not shown). Although MG116025 is included among Wave 2 atypical El Tor strains, the CT that it produces is the El Tor-type toxin (*ctxB3*). Approximately equal amounts of CT were produced from MG116025 and the reference O395 at 30°C, whereas CT production at 37°C was negligible (Figure 2C).

In summary, CT production varies between Wave 2 El Tor strains, and approximately twice as much CT can be produced from a derivative of B33 that contains the *toxT*-139F allele versus the reference classical biotype strain. Although MG116025 is a Wave 2 strain, its pattern of CT production was similar to that of Wave 1 strains, because CT was produced at 30°C. The function of VC1589, and thus the capacity for neutral fermentation, did not affect CT production in Wave 2 strains.

### Cholera Toxin Production in Wave 3 Strains

Two Wave 3 atypical El Tor strains were examined. IB4122 was isolated from a cholera outbreak in Northern Vietnam in 2009, and IB5230 was isolated from the 2010 cholera outbreak in Haiti (Nguyen et al., 2009; Chin et al., 2011). Both strains originally contained the *toxT*-139Y allele.

#### Derivatives of IB4122 That Contain the *toxT*-139F Allele

**FIGURE 3:**
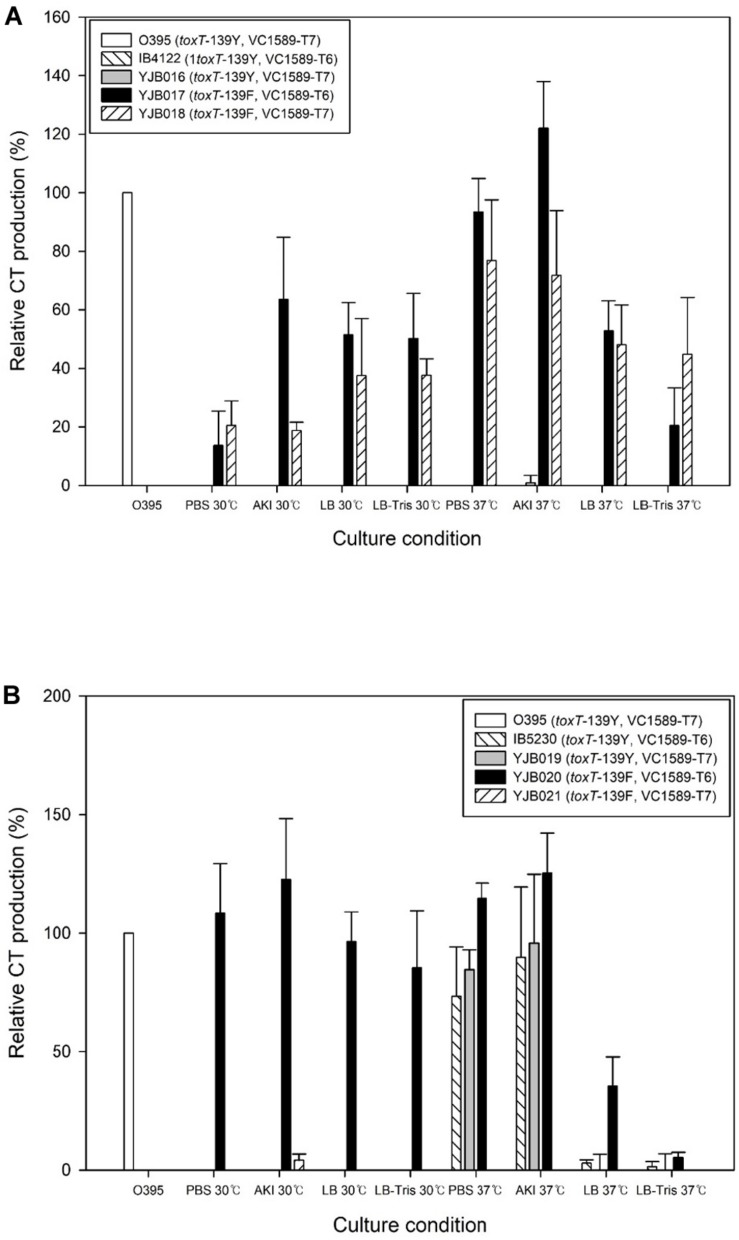
CT production in Wave 3 El Tor strains and their derivatives. **(A)** IB4122 and one of its derivatives (YJB016) that contained the *toxT*-139Y allele produced no CT. The IB4122 derivatives YJB017 and YJB018 that contained the *toxT*-139F allele produced CT under laboratory conditions. The optimal conditions for CT production were cultured at 37°C in AKI media. The function of the VC1589 was not associated with significant differences in CT production in YJB017 or YJB018. **(B)** CT production in IB230 is unique compared with other El Tor strains. IB5230 that contained the *toxT*-139Y allele and nonfunctional VC1589 produced CT at 37°C in PBS-buffered LB and AKI media, whereas other El Tor biotype strains that contained *toxT*-139Y did not produce CT. Moreover, YJB020, a derivative of IB5230 that contained the *toxT*-139F allele and nonfunctional VC1589, produced CT, and YJB021 that contained *toxT*-139F and functional VC1589 did not produce CT.

The CT that was produced by this strain was the classical type toxin (*ctxB1*). Approximately equal amounts of CT, with the reference strain, were produced by YJB017 (*toxT*-139F, VC1589-T6) and YJB018 (*toxT*-139F, VC1589-T7), which contained the *toxT*-139F allele, at 37°C in AKI media; slightly more CT was generated at 37°C than at 30°C (Figure 3A).

#### IB5230 and Derivatives of IB5230 That Contain the *toxT*-139F Allele

IB5230 is considered a hypervirulent strain due to its elevated CT production and colonization of the intestine (Satchell et al., 2016; Ghosh et al., 2019). The CT that was produced by this strain is *ctxB7*, which differs from the classical type *ctxB* (*ctxB1*) at amino acid residue 20. However, the secreted form of CT from this strain is the same as classical type CT, because the first 21 amino acids are removed during secretion (Sanchez and Holmgren, 2008).

Notably, although IB5230 harbors the *toxT*-139Y allele, CT production in PBS-buffered LB or AKI media at 37°C was similar to that by the reference strain (Figure 3B). The function of VC1589 did not affect the production of CT under these culture conditions as similar amount of CT was produced from strain YJB19 (*toxT*-139Y, and VC1589-T7), implying that IB5230 can be applied directly to the production of CT without alteration in *toxT*.

Another unique characteristic of IB5230 is that the function of VC1589, and hence the capacity for neutral fermentation, interfered with CT production when the *toxT*-139Y allele was replaced with *toxT*-139F. A derivative of IB5230, YJB20, containing the *toxT*-139F allele and nonfunctional VC1589, produced as much CT as the reference strain, similar to other strains that contain *toxT*-139F; however, YJB021, which harbors the *toxT*-139F allele and functional VC1589, did not produce CT under any culture condition (Figure 3B). Although the function of VC1589 affects the fermentation switch in media that has been supplemented with additional glucose, it appears that the operative metabolic pathway also influences toxin production in *V. cholerae*. More detailed studies of CT production in IB5230 and its derivatives might identify additional important characteristics that underlie the hypervirulent nature of this strain.

### Enhanced Cholera Toxin Production in a *V. cholerae* Classical Biotype Strain

**FIGURE 4:**
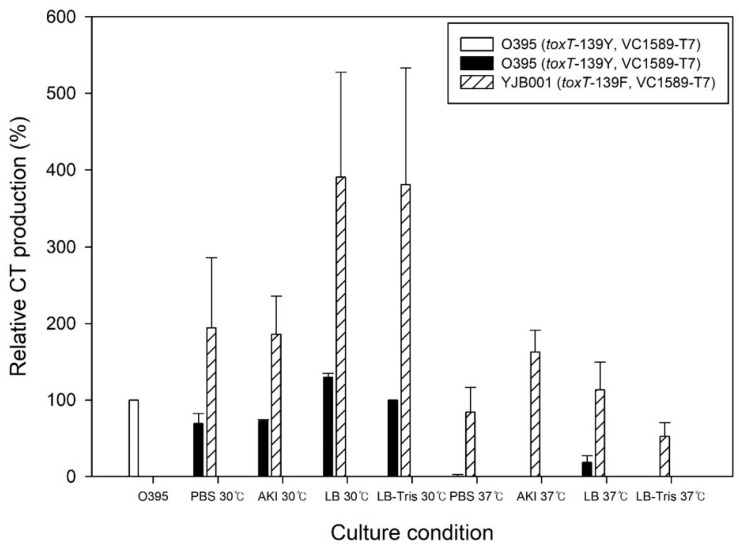
CT production in a classical biotype strain O395 and its derivative that contains the *toxT*-139F allele. YJB001, a derivative of O395 that contained the *toxT*-139F allele, produced up to 200% CT in PBS-buffered LB and AKI media and up to 400% CT in LB and Tris–buffered LB at 30°C relative to the reference. O395 did not produce detectable amounts of CT at 37°C, but YJB001 produced CT in amounts comparable with the reference at 37°C.

A derivative of O395, YJB001, that contained the *toxT*-139F allele produced 400% more CT than O395 that harbored the *toxT*-139Y allele when cultured in regular LB media at 30°C and under agglutinating conditions (Figure 4). More CT was produced at 30°C than 37°C. In conclusion, the *toxT*-139F allele induces classical *V. cholerae* strains to produce more CT under laboratory conditions.

## Discussion

The toxigenesis of *V. cholerae* has received much attention with regard to understanding the virulence of bacteria (Clemens et al., 2017). A protocol for generating CT under laboratory conditions has been developed for classical biotype strains—described as “agglutinating conditions” (DiRita et al., 1996). Several special culture methods have been developed to promote CT production in El Tor biotype strains: the use of a bi-phasic culture method for CT production under the AKI condition (Sanchez et al., 2004); increases in the surface area-to media-volume ratio to stimulate CT production under laboratory conditions (Iwanaga et al., 1986); and a shallow static culture method for CT production (Cobaxin et al., 2014).

However, these culture methods are not appropriate for monitoring toxigenesis in bacteria or large-scale CT production (Cobaxin et al., 2014). In the present study, we developed laboratory culture conditions under which El Tor biotype *V. cholerae* strains produced CT in a simple single-phase culture.

The expression of CT and TCP is stimulated by ToxT, an AraC/XylS-type transcriptional regulator (Yu and DiRita, 2002; Taylor et al., 2004). *toxT* expression is tightly regulated by ToxR/ToxS and TcpP/TcpH, which in turn are controlled by various regulatory proteins and stimuli in the intestinal environment (Sanchez and Holmgren, 2008). We identified a *V. cholerae* Wave 2 atypical El Tor strains, MG116025, that contains a point mutation in *toxT* (Phe at amino acid position 139, instead of Tyr in most other strains) (Kim et al., 2014, 2017). Amino acid position 139 of ToxT belongs to the N-terminal domain (amino acids 1–160), which has been implicated in regulation of binding to the cognate DNA sites of ToxT (Thomson and Withey, 2014; Thomson et al., 2015). The expression of TCP and CT is higher in this strain, and we anticipated the *toxT*-139F allele to be useful for examining and producing CT in *V. cholerae* strains (Kim et al., 2017). As expected, CT production in El Tor strains was stimulated by the same point mutation in *toxT*, but the toxin production varies by strain. These results also imply that the expression of TCP and CT is constitutively stimulated by pre-existing ToxT but not by newly synthesized ToxT.

We determined the optimum single-phase culture methods for CT production in El Tor strains. A representative Wave 1 El Tor biotype strain, N16961, produces CT under specific culture conditions (Lee et al., 2012; Satchell et al., 2016). However, this strain failed to produce CT under regulation of the *toxT*-139F allele. When *toxT*-139F was introduced, another Wave 1 strain, T19479, generated approximately 40% of the CT that was produced by the classical biotype strain O395 under agglutinating conditions. CT production varies between atypical El Tor strains. Atypical El Tor strains tend to produce more CT than prototype El Tor biotype strains under regulation of the *toxT*-139F allele. A notable characteristic of classical and Wave 1 El Tor strains is that they produce more CT at 30°C than at 37°C, whereas Wave 2 and 3 atypical El Tor strains synthesize similar amounts of CT at both temperatures. This might be an important factor for population changes or the Waves in El Tor biotype strains within the seventh cholera pandemic.

Recently, we found that Wave 2 and 3 atypical El Tor strains lack neutral fermentation due to the loss of function of the acetolactate decarboxylase that is encoded by VC1589 (Lee et al., 2020). This change in phenotype did not affect CT production in most El Tor strains, except for the hypervirulent 2010 Haiti cholera outbreak strain that produced CT only when neutral fermentation was disrupted (Chin et al., 2011). However, acidic fermentation occurred when glucose was sufficiently supplied in the media, and the bacteria did not experience much difference in growth without fortified glucose, regardless of whether the VC1589 was functional. More detailed follow-up studies are required to determine the link between the fermentation pathways and toxigenesis in this strain, especially because it is considered a hypervirulent *V. cholerae* strain.

We also found that CT production increased approximately four-fold under agglutinating conditions in classical biotype strains when the *toxT*-139Y allele was replaced by *toxT*-139F. The optimal culture conditions for CT production by classical biotype strains that contained the *toxT*-139F allele were regular LB media at 30°C and agglutinating conditions.

Cholera toxin has tremendous potential as a mucosal vaccine adjuvant (Terrinoni et al., 2019). Efforts to develop mutated CT to avoid enterotoxicity when used as a vaccine adjuvant are ongoing. The method that we have established to mass-produce CT might support the development of a potent CT-based vaccine adjuvant (Lebens et al., 2016).

## Data Availability Statement

All datasets generated for this study are included in the article/Supplementary Material.

## Author Contributions

EK and DK designed the research. YB, DL, EK, JL, and YY performed the research. GN contributed the bacterial strains. YB, DL, EK, JL, and YY analyzed the data and created figures. EK and DK wrote the manuscript. All authors reviewed the manuscript.

## Conflict of Interest

The authors declare that the research was conducted in the absence of any commercial or financial relationships that could be construed as a potential conflict of interest.

## Supplementary Material

FIGURE S1 Immunoblot analysis of cholera toxin production in *V. cholerae* strains. As a representative, Western blot analysis of CT in strains MG116025 and one of its derivatives YJB014 is shown in this figure. Bacterial culture that contains approximately 5 × 10^7^ cells were loaded onto each lane. Lanes M: Protein molecular weight marker, Lane 1: O395 reference, Lanes 2, 3: MG116025 cultured in PBS-buffered LB at 30°C, Lanes 4, 5: MG116025 cultured in PBS-buffered LB at 37°C, Lanes 6, 7: YJB014 cultured in LB at 30°C, Lanes 8, 9: YJB014 cultured in LB at 37°C. White and black arrows indicate CTA and CTB, respectively.

FIGURE S2 CT production in Wave 1 strains N16961 and its derivatives. N16961 has been shown to produce CT under the AKI conditions: however, no detectable CT was produced from N16961 or its derivatives that contained *toxT*-139F allele.
