# Supplementary figures and images for: Cholera Toxin Production in Vibrio cholerae O1 El Tor Biotype Strains in Single-Phase Culture
Source: Front Microbiol. 2020 May 5;11:825. doi: 10.3389/fmicb.2020.00825 (PMC7214932; doi:10.3389/fmicb.2020.00825)

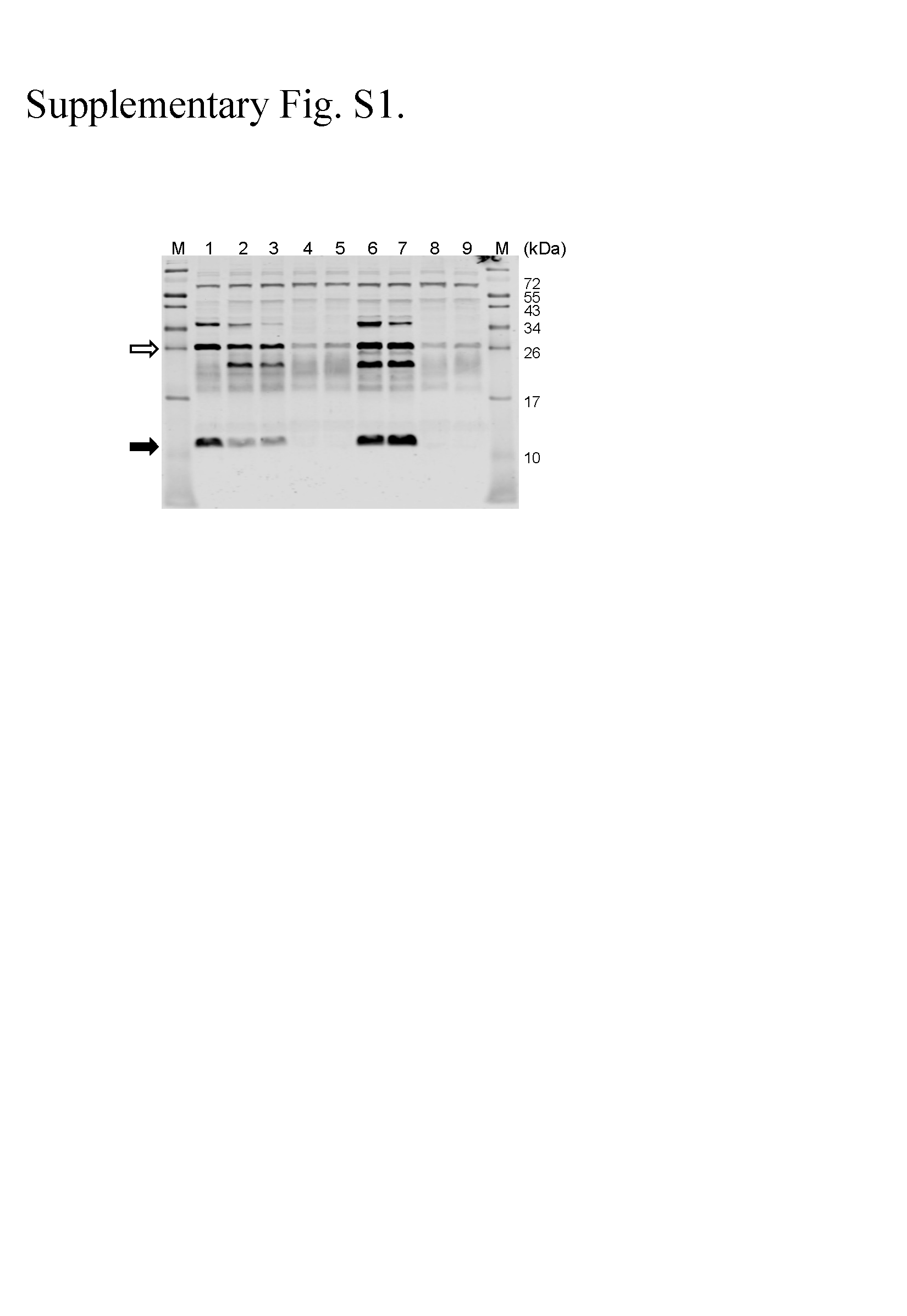

Supplement: FIGURE S1 — Immunoblot analysis of cholera toxin production in V. cholerae strains. As a representative, Western blot analysis of CT in strains MG116025 and one of its derivatives YJB014 is shown in this figure. Bacterial culture that contains approximately 5 × 10^7^ cells were loaded onto each lane. Lanes M: Protein molecular weight marker, Lane 1: O395 reference, Lanes 2, 3: MG116025 cultured in PBS-buffered LB at 30°C, Lanes 4, 5: MG116025 cultured in PBS-buffered LB at 37°C, Lanes 6, 7: YJB014 cultured in LB at 30°C, Lanes 8, 9: YJB014 cultured in LB at 37°C. White and black arrows indicate CTA and CTB, respectively. [file Image_1.tiff]

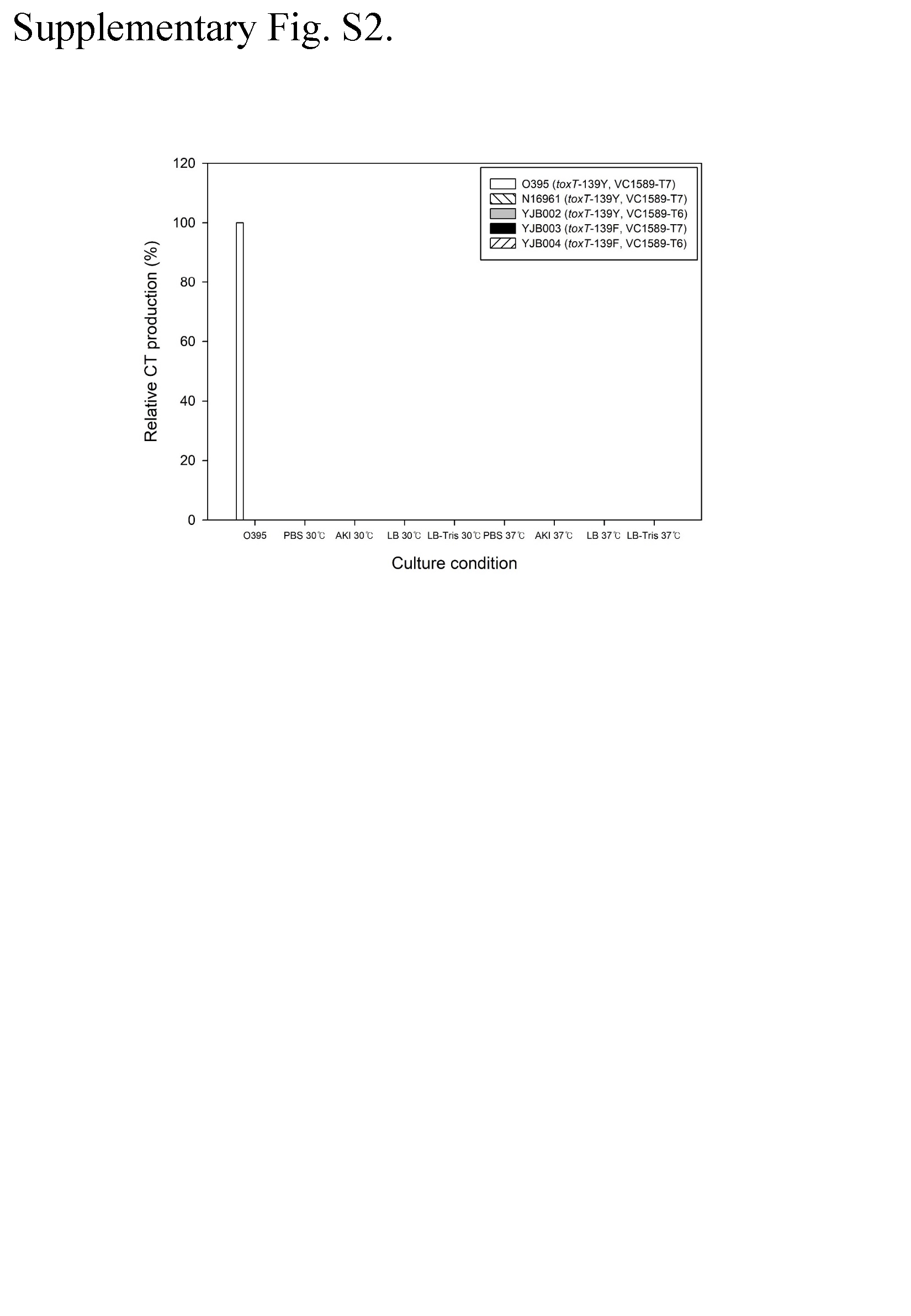

Supplement: FIGURE S2 — CT production in Wave 1 strains N16961 and its derivatives. N16961 has been shown to produce CT under the AKI conditions: however, no detectable CT was produced from N16961 or its derivatives that contained toxT-139F allele. [file Image_2.tiff]
